# Final-Year Dental Undergraduate Attitudes towards Specialisation

**DOI:** 10.3390/dj4030026

**Published:** 2016-08-10

**Authors:** James Puryer, Veselina Kostova, Alisa Kouznetsova

**Affiliations:** School of Oral and Dental Sciences, Bristol Dental Hospital, Lower Maudlin Street, Bristol BS1 2LY, UK; vessy@protonmail.com (V.K.); alisa.kouznetsova@hotmail.com (A.K.)

**Keywords:** UK, undergraduate, attitudes, specialisation

## Abstract

The aim of this study was to explore the attitudes towards postgraduate specialisation of final-year students at one UK dental school and to identify any possible influencing factors. A cross-sectional survey of all 73 final-year students using an anonymous self-report questionnaire was carried out. The response rate was 79%, of which nearly two-thirds were female. Age, ethnicity and parental occupation did not have an effect on the intention to specialise, although student gender did, with more females not wishing to specialise (*p* = 0.006). Having a ‘talent in the field’ had the largest positive influence on pursuing a specialist career (37.9% of responses), whilst the length of time needed to obtain a specialist qualification was seen as the largest negative influence (41.1% of responses). The two most popular subjects were Restorative Dentistry and Orthodontics with 24.1% and 20.7% of students listing them as their first choices, respectively. Further research could be carried out to determine if the intentions of UK undergraduates to specialise will meet the increasing specialist oral health needs of the population and which could ultimately influence future dental workforce planning.

## 1. Introduction

In the United Kingdom (UK), dental undergraduates undergo a 5-year programme of study at one of 17 dental schools which, upon successful completion, allows them to register with the General Dental Council (GDC). Each year, there are approximately 1300 graduates [1] who then undertake a year of Dental Foundation Training (DFT) within a general practice setting. Subsequently, there are many avenues for career development open to them. Usually, this will involve returning to an academic institution or hospital-based unit in order to gain further knowledge and skills within a specialist area of dentistry. This can lead to further registrable qualifications and eventual recognition by the General Dental Council (GDC) through the specialist list. Currently, there are approximately 41,000 dentists on the UK register [2] with just over 10% of them being on a specialist list. The 13 dental specialties that are currently recognised by the General Dental Council and the number of registrants per specialty are shown in Table 1.

A recent study found that a large proportion (71%) of UK undergraduates expressed a desire to specialise following graduation, with Orthodontics being the most popular intended specialty [3]. This supports an earlier UK study which found that 83% of recent graduates were either ‘certain of’ or ‘considering’ pursuing a specialist career, [4] although for this group of young dentists, Restorative dentistry was the most preferred intended pathway. For these recent graduates, the ‘length of training’, ‘cost’ and ‘disruption of life’ were reported to be the three greatest barriers to further specialisation. Further positive attitudes towards specialisation were found in final-year students from four Iranian dental schools [5].

The factors that influence UK undergraduate dental students’ specific career choices have not been investigated comprehensively, although studies have been carried out in other countries. A 2007 study [6] investigated the views of Japanese, Canadian and Thai undergraduates and found that 48.4% of Canadian, 38.8% of Japanese and 39.3% of Thai students favoured specialisation. Factors including high student debt and expensive tuition fees influenced Canadian students towards general dentistry, whilst inheriting the family dental practice was probably the most influential factor steering Japanese students away from specialisation. For Thai students, the main reason behind choosing a specialty career was improved financial prospects. Amongst Canadian and Japanese participants, Orthodontics was the most preferred subject, whereas Thai students’ preferred choice was Oral Surgery. A subsequent study [7] investigated tendencies for specialisation amongst final year Japanese and Swedish dental students. Only 16.7% of Japanese compared to 37.2% of Swedish students were keen to specialise, echoing the earlier study finding that the majority of Japanese students intend to stay within general practice. The Swedish students’ main motive for choosing a specialist career was increased financial prospects. In both countries approximately one-third of those who had indicated they would like to specialise had not yet chosen their specialty, although of those that had, Orthodontics was again the most preferred subject amongst Japanese students, and Oral Surgery the most preferred subject amongst Swedish students.

In the USA, it was found that a dental undergraduate’s “possession of special skills or talent” was the main reason for wanting to specialise rather than any financial factor, [8] and again, Orthodontics was the most preferred subject. This study also concluded that gender was not an important determinant when it came to choosing a speciality, which is consistent with previous studies [4,9]. However, more recent research [10] carried out amongst fourth year dental students at another school within the USA found that gender did have an impact on the choice of specialty, and that males were seven times more likely to advance into an Oral and Maxillofacial Surgery field, whereas females were four times more inclined to study Paediatric Dentistry. This study found that ’enjoyment of providing care’ was the major influencing factor for choosing any postgraduate educational direction. Findings of the Annual American Dental Education Association [11] whose research involved senior dental students from all dental schools across the USA also reported Orthodontics to have been the most popular specialty for dental students from 1995 to 2004, followed by Oral and Maxillofacial Surgery and Paediatric Dentistry. Not surprisingly these three specialties were also the most commonly offered as postgraduate courses by both American and Canadian dental schools [12].

Whilst research has been carried out into UK dental undergraduates career plans including their intentions to specialise [3,13], little is known about what influences their decision to specialise in a particular branch of dentistry.

## 2. Aim

To explore the attitudes towards postgraduate specialisation of final year students at one UK dental school and to identify any possible influencing factors.

## 3. Method

A cross-sectional survey of all final year dental undergraduates (*n* = 73) studying at the University of Bristol was carried out. The questionnaire (Appendix A) was designed to investigate the students’ backgrounds and their perspectives towards postgraduate specialisation as well as factors that would discourage/influence their choices. It consisted of two sections: four questions regarding the students’ background and six questions regarding their attitudes towards postgraduate specialisation. A closed-style of questioning was used, with all of the questions being ‘tick-box’ responses to aid speed of completion, as well making quantitative statistical analysis possible.

The questionnaire was distributed to the students at the end of one of their compulsory lectures along with an information sheet which explained the nature of the survey and the anonymity of responses. Participation was non-compulsory, and following voluntary completion of the questionnaire, the questionnaires were deposited in a box as students left the lecture theatre.

Full ethical approval from the Faculty of Medicine and Dentistry Committee for Ethics was obtained prior to the study.

SPSS 19 was used to carry out the statistical analysis of the results.

## 4. Results

Questionnaires were completed by *n* = 58 undergraduates, out of a possible *n* = 73. All of the questionnaires were completed in full and were included in the study, which gave a usable response rate of 79%. The majority (60.3%) were female.

**Demographics**: The ages and ethnicity of the respondents are shown in Table 2 and Table 3. The majority of students were aged 20–23 years and more than half of students were White British.

**Desire to pursue a specialty career**: Thirty eight per cent (*n* = 22) of students responded that they wished to pursue a specialist career versus 14% (*n* = 8) who responded that they did not. 48.3% (*n* = 28) expressed uncertainty regarding their future desire to specialise, although the majority of this group still specified their preferred first and second specialty choices. Age (*p* = 0.083) and ethnicity (*p* = 0.264) were not found to have an effect on intention to specialise.

**Figure 1:**
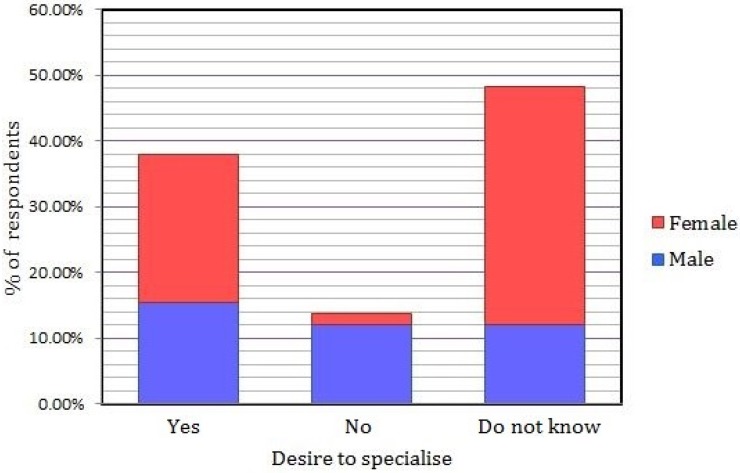
Percentage of respondents by gender and desire to pursue a specialist career.

**Gender**: 39.1% (*n* = 9) of males and 37.1% (*n* = 13) of females indicated they would like to pursue a specialist career. A difference was found with 30.4% (*n* = 7) of males and 2.9% (*n* = 1) of females not wishing to specialise (*p* = 0.006). Figure 1 shows the percentage of respondents by gender and their intention to pursue a specialist career.

**Parental Occupation vs. desire to pursue a specialist career**: To aid statistical analysis, it was assumed that each individual that took part in the study had a household consisting of two parents, who could be grouped into one of the three professional groups (professional and managerial/technical, skilled (non-manual)/skilled (manual), and partly skilled/unskilled). Statistical analysis shows that choice of whether or not participants want to specialise is not dependent on their parental occupation (*p* = 0.663 and *p* = 0.450 for the first and second parent respectively).

**First choice of specialty**: The most popular first career choices amongst final year dental students were Restorative Dentistry (24.1%), Orthodontics (20.7%) and Oral Surgery (13.8%). None of the students selected Oral medicine or Oral Microbiology as a first career choice.

**Second choice of specialty**: Oral Surgery (20.7%) followed by Orthodontics (17.2%) and Endodontics (17.2%) were listed as the most desired second choice of specialty. Restorative Dentistry was selected as second choice of specialty by 8.6% (5/58) of students (Table 4). When combining the first and second choices, the most popular intended subjects were Orthodontics (37.9%) and Restorative Dentistry (32.7%).

**Gender within first specialty choice**: Restorative Dentistry was first choice for almost twice as many males 64.3% than for females 35.7%. More females 83.3% than males (16.7%) reported Orthodontics to be their first choice of specialty. The fields of Paediatric Dentistry, Prosthodontics, Dental Public Health, Oral and Maxillofacial Pathology and Dental and Maxillofacial Radiology were only chosen by female students, whilst the fields of Endodontics and Periodontics were selected by males only. No gender differences were found amongst Special Care Dentistry and Oral Surgery.

**Factors influencing decision to specialise**: By far the most common reason indicated for specialising was ‘talent in the field’. ‘Reward’, ‘financial reasons’ and ‘further studies’ were chosen as the second most selected motives for undertaking specialist training (Table 5).

**Factors discouraging from specialisation**: Almost one-half of dental students (41.4%) identified the length of acquiring a postgraduate degree as a deterring factor from becoming a specialist. The further studying involved in specialisation and expenses associated with it were an obstacle for 24.1% and 22.4% of students respectively (Table 6).

**Encouragement to pursue a specialist career**: Almost two-thirds (65%) of students felt they had received sufficient encouragement and support from the university to be able to make a decision on specialisation.

## 5. Discussion

This study reports the attitudes towards postgraduate specialisation among final year undergraduate dental students with respect to their gender, ethnic group and parental occupation. The questionnaire response rate of 79% is similar to other questionnaire-based studies in the same research field and increases the validity of the study [14]. The demographics of respondents are representative of the whole cohort of students in terms of their gender, age and ethnicity, although it is possible that there is some selection bias amongst the respondents. However, the high response rate will help to minimise any possible selection bias.

Almost half of dental undergraduates were undecided whether or not to pursue a specialist career, which is higher than previously quoted figures [3,6,7,13]. This could be accounted to the fact that encouragement from the educational institution plays an important role in the decision making process and approximately one-third of students in this study did not feel they had sufficient encouragement and exposure to dental specialties to make this decision yet. It has been suggested that a student is 5.93 times more likely to specialise if they have received encouragement compared to a student who has not [9]. However, it must be remembered that dental undergraduates have yet to experience professional life after qualification, and so it would be understandable that many would still be uncertain as to the career path that they wish to follow.

Although the majority of Bristol dental students were indecisive on specialisation, over one-third of respondents were intending to pursue a postgraduate career which was in line with previous findings [6,15]. The most popular reason for wanting to specialise given by students in this current study (having talent in a particular field) is consistent with the reason given by dental students in the USA, [8] and also by junior doctors within the UK [16]. This is encouraging as it may lead to enthusiastic specialists with high levels of clinical skills which would be to the benefit of patients.

For this current year of dental undergraduates, the time consuming nature of acquiring a postgraduate qualification was selected by majority of students as a deterring factor, supporting earlier research [4]. A dentist within the UK is not eligible to apply for specialist training until at least 2 years post-qualification, and this training can take a further 5 years of study. Some dentists, particularly those with families, will need to maintain a healthy work-life balance and may not be able to invest the time needed to pursue a specialist career. It is not surprising that the amount of extra studying needed was the second most popular deterring factor, as dental students will have already spent approximately 20 years in full-time education, and for some, the prospect of so many more years studying was not encouraging.

In contrast to previous studies [4,9], this current study found that gender had an influence on decision to specialise, and significantly, twice as many females compared to males were undecided on specialisation. This is understandable as female dentists, in addition to working and studying, are often committed to bringing up children, with, on average 71% of female dentists reported as having children [17]. Childcare needs will influence the future work patterns of female dentists [13]. In addition, more female than male dental students within the UK expect to take more time out of their future careers to concentrate on childcare [3].

Ethnicity was not found to have any influence on students’ intention to specialise which is consistent with other recent findings [3].

There was no evidence found that parental occupation had an influence on the respondents’ decision to specialise. These findings could lead to the assumption that dental students that took part in the study were independent and mature individuals that were not guided by their parents’ work experience. Dentistry is a five year professional course that is designed to equip people with leadership skills and make rational decisions on their own, and this is encouraging.

Within the UK, the specialty of Restorative Dentistry comprises the three mono-specialties of Endodontics, Periodontics and Prosthetics. In this study, these were treated as four separate specialties so as to allow comparison with previous studies. Restorative Dentistry was found to be the most popular subject to study within this current group of students which supports the findings of an earlier UK study [13] of dental undergraduates from a London dental school. One possible explanation for this is that Restorative Dentistry takes up a large proportion of the undergraduate curriculum, and as ‘having talent in the field’ was the most common reason expressed for choosing a specialty, it would not be unreasonable to assume that students were likely to realise their talent and potential in a subject in which they devote considerable time as an undergraduate. In addition, these undergraduates may perceive that there will be strong patient demand for specialist Restorative dental services within private dental practice, such that it is the potential financial reward of this subject that is appealing. Other subjects such as Oral Microbiology may be perceived as having limited demand for services and limited employment opportunities. Orthodontics was the second most popular choice of specialty with current respondents which is in contrast to other studies, all of whom reported that Orthodontics was the most preferred specialty [3,6,8,11,18]. Undergraduates at Bristol only study Orthodontics in Years 4 and 5, (compared to Restorative Dentistry which is studied in Years 2 to 5) which supports the argument that exposure time to a particular subject may have an effect on aspirations to specialise in that subject. The field of orthodontics was ranked high in terms of improving dentists’ and patients’ quality of life as well as giving financial prosperity in a previous study [10], and so it was not surprising that it is one of the most preferred career choices. Oral Surgery, although only being ranked third in the current study, was the most preferred postgraduate career for Nordic dental students [19] and Swedish dental students [7].

All of the respondents who selected Paediatric Dentistry as a first career choice were female. This was in line with the findings of studies carried out among American [10] and Bulgarian [20] dental students and dentists respectively, who concluded Paediatric Dentistry to be a female dominated profession. Previous studies [21,22] have reported an increase in the overall number of female UK dentists specialising, and also gender differences within specialties. This, along with the fact that the number of females within the UK dental workforce is increasing, may have future impact on access to dentistry and specialist services.

Over one-third (35%) of respondents felt that they had not received sufficient information and encouragement whilst as an undergraduate to help them decide upon a possible specialist career. It has been shown [9] that undergraduate experience plays a major role in influencing a student’s decision to undertake postgraduate study, along with encouragement received from dental school staff, partners and family. It is likely that an individual student choosing to obtain postgraduate qualifications is highly influenced by the relationship with the specialist clinical staff they encounter as during their undergraduate studies, highlighting the important ‘role model’ facet to academic teaching staff.

This study does have some limitations. Dental undergraduates at a single UK dental school may not be representative of all UK undergraduates, and so it would be incorrect to assume that these results could be generalised, although this would also apply to other previous similar studies. In addition, it must be remembered that when looking at the views of undergraduates regarding future specialisation, it may be more appropriate to consider these as ‘aspirations to specialise’ rather than the number of undergraduates who will make this a reality. Considering that just over 10% of UK dentists are registered specialists, it is likely that without a significant increase in the number of available training places, there will be strong competition for these places, leaving some dentists unable to fulfil their intended career pathways. In addition, an individual student’s views on specialisation may differ following qualification once they have experienced practising life, and their family and career plans may ultimately change. Wider research could be carried out to investigate the intentions to specialise of both UK undergraduates and recent Dental Foundation Trainees, along with those qualifying overseas, and it may be appropriate to take this into account when planning the future UK dental workforce. There is evidence that the UK needs a far greater number of specialists in order to meet the dental needs of the population [23,24], and the encouraging results from this study in that a large proportion of current undergraduates intend to specialise will hopefully help to ensure that the ever-evolving oral health needs of the UK population are met.

## 6. Conclusions

This study shows that a large number of undergraduates had an intention to specialise, with Restorative Dentistry and Orthodontics being the most popular intended subjects, although many were still undecided at this stage. Age, ethnicity and parental occupation did not have any influence on intentions to specialise, although student gender did. Having a ‘talent in the field’ had the largest positive influence on pursuing a specialist career, whilst the length of time needed to obtain a specialist qualification was seen as the largest negative influence. The results of this study are representative of the views of final year undergraduates at one dental school in the United Kingdom, and further studies could be undertaken to identify whether the intentions of undergraduates are likely to meet the increasing specialist dental needs of the UK population, and to take this into account when planning the future dental workforce.

## Figures and Tables

**Table 1:** Dental specialties recognised by the General Dental Council (GDC) and number of registrants (October 2015).

Specialty	Number of Registrants
Orthodontics	1373
Oral Surgery	754
Prosthodontics	450
Periodontics	368
Restorative Dentistry	317
Special Care Dentistry	311
Endodontics	277
Paediatric Dentistry	240
Dental Public Health	117
Oral Medicine	70
Oral & Maxillofacial Pathology	30
Dental & Maxillofacial Radiology	27
Oral Microbiology	8
TOTAL	4342

**Table 2:** The ages of respondents.

Age Group	Percentage of Students
20–23 years	65.5%
24–26 years	19.0%
27–29 years	3.4%
30+ years	12.1%

**Table 3:** The ethnicity of respondents.

Ethnicity	Percentage of Students
White British	53.5%
White ‘Other’	0%
Mixed/multiple ethnic groups	6.9%
Asian/Asian British	29.3%
Black/African/Caribbean/Black British	1.7%
Other ethnic group	8.6%

**Table 4:** First and second choices of specialty.

Specialty	First Choice (*n* = 58)	Second Choice (*n* = 58)
Special Care Dentistry	3.4%	1.7%
Oral Surgery	13.8%	20.7%
Orthodontics	20.7%	17.2%
Paediatric Dentistry	12.1%	8.6%
Endodontics	6.9%	17.2%
Periodontics	3.4%	3.4%
Prosthodontics	1.7%	0%
Restorative Dentistry	24.1%	8.6%
Dental Public health	1.7%	3.4%
Oral Medicine	0%	6.9%
Oral Microbiology	0%	0%
Oral & Maxillofacial Pathology	3.4%	3.4%
Dental & Maxillofacial Radiology	3.4%	1.7%
Missing Responses	5.2%	6.9%

**Table 5:** Factors influencing decision to specialize.

Factor	Percentage of Students (*n* = 58)
Family & friends’ expectations	10.3%
Social status	1.7%
Reward	13.8%
Talent in the field	37.9%
Financial reasons	13.8%
Lack of existing specialists in field	1.7%
Further studying	13.8%
No response	6.9%

**Table 6:** Factors discouraging decision to specialize.

Factor	Percentage of Students (*n* = 58)
Time consuming	41.1%
Too expensive	22.4%
No prospects/need	3.4%
Too competitive	8.6%
Further studying	24.1%

## Appendix A

The survey questionnaire.
